# Comment on “The Role of Overweight and Obesity in In Vitro Fertilization Outcomes of Poor Ovarian Responders”

**DOI:** 10.1155/2015/318902

**Published:** 2015-10-05

**Authors:** Raoul Orvieto

**Affiliations:** ^1^Infertility and IVF Unit, Department of Obstetrics and Gynecology, Chaim Sheba Medical Center at Tel Hashomer, Ramat Gan 52621, Israel; ^2^Sackler Faculty of Medicine, Tel Aviv University, Tel Aviv, Israel

We read with interest the study by Vural et al. [1], who aimed to retrospectively explore the effects of overweight and obesity on IVF outcomes of poor ovarian responders (POR). While the oocyte maturity, total gonadotropin dose-duration, and cycle cancellation rates were similar, they observed significantly lower fertilization and clinical pregnancy rates in obese compared to normal and overweight POR. Surprisingly, our previous study, which was the first to challenge the relationship between obesity and POR, went unnoticed [2]. While examining whether body mass index (BMI) may influence IVF outcome, we could demonstrate that obese patients had a significantly higher prevalence of poor responders as compared to nonobese patients, required longer stimulation, used significantly more gonadotropin ampoules, had lower peak estradiol levels, and showed significantly lower fertilization rate. Moreover, in agreement with Vural et al. [1], obese poor responder patients achieved a significantly lower pregnancy rate compared to nonobese poor responders. We therefore concluded that while the likelihood of poor responders is increased among obese women, reasonable conception rates were achieved in nonobese poor responders, which were comparable to nonobese or normal responder patients. Moreover, until sufficient prospective studies on the effect of weight reduction on IVF outcomes of POR women appear, we recommended that POR women with obesity should receive meticulous counsel for weight reduction programs before an IVF trial.

## References

[1] Vural F., Vural B., Çakıroğlu Y. (2015). The role of overweight and obesity in in vitro fertilization outcomes of poor ovarian responders. *BioMed Research International*.

[2] Orvieto R., Meltcer S., Nahum R., Rabinson J., Anteby E. Y., Ashkenazi J. (2009). The influence of body mass index on in vitro fertilization outcome. *International Journal of Gynecology and Obstetrics*.

